# Design and Testing of a Custom Melanoma Next Generation Sequencing Panel for Analysis of Circulating Tumor DNA

**DOI:** 10.3390/cancers12082228

**Published:** 2020-08-10

**Authors:** Russell J. Diefenbach, Jenny H. Lee, Alexander M. Menzies, Matteo S. Carlino, Georgina V. Long, Robyn P. M. Saw, Julie R. Howle, Andrew J. Spillane, Richard A. Scolyer, Richard F. Kefford, Helen Rizos

**Affiliations:** 1 Department of Biomedical Sciences, Faculty of Medicine, Health and Human Sciences, Macquarie University, Sydney, NSW 2109, Australia; russell.diefenbach@mq.edu.au (R.J.D.); jenny.lee@mq.edu.au (J.H.L.); 2 Melanoma Institute Australia, The University of Sydney, Sydney, NSW 2065, Australia; alexander.menzies@sydney.edu.au (A.M.M.); matteo.carlino@sydney.edu.au (M.S.C.); georgina.long@sydney.edu.au (G.V.L.); robyn.saw@melanoma.org.au (R.P.M.S.); julie.howle@sydney.edu.au (J.R.H.); andrew.spillane@sydney.edu.au (A.J.S.); richard.scolyer@health.nsw.gov.au (R.A.S.); richard.kefford@mq.edu.au (R.F.K.); 3 Sydney Medical School, The University of Sydney, Sydney, NSW 2006, Australia; 4 Department of Medical Oncology, Northern Sydney Cancer Centre, Royal North Shore Hospital, Sydney, NSW 2065, Australia; 5 Crown Princess Mary Cancer Centre, Westmead and Blacktown Hospitals, Sydney, NSW 2145, Australia; 6 Charles Perkins Centre, The University of Sydney, Sydney, NSW 2006, Australia; 7 Department of Melanoma and Surgical Oncology, Royal Prince Alfred Hospital, Sydney, NSW 2050, Australia; 8 Breast and Melanoma Surgery Department, Division of Surgery, Royal North Shore Hospital, Sydney, NSW 2065, Australia; 9 Department of Tissue Pathology and Diagnostic Oncology, Royal Prince Alfred Hospital and New South Wales Health Pathology, Sydney, NSW 2050, Australia; 10 Department of Clinical Medicine, Faculty of Medicine, Health and Human Sciences, Macquarie University, Sydney, NSW 2109, Australia

**Keywords:** Melanoma, circulating tumor DNA, targeted sequencing, custom panel

## Abstract

Detection of melanoma-associated mutations using circulating tumor DNA (ctDNA) from plasma is a potential alternative to using genomic DNA from invasive tissue biopsies. In this study, we developed a custom melanoma next-generation sequencing (NGS) panel which includes 123 amplicons in 30 genes covering driver and targetable mutations and alterations associated with treatment resistance. Analysis of a cohort of 74 stage III and IV treatment-naïve melanoma patients revealed that sensitivity of ctDNA detection was influenced by the amount of circulating-free DNA (cfDNA) input and stage of melanoma. At the recommended cfDNA input quantity of 20 ng (available in 28/74 patients), at least one cancer-associated mutation was detected in the ctDNA of 84% of stage IV patients and 47% of stage III patients with a limit of detection for mutant allele frequency (MAF) of 0.2%. This custom melanoma panel showed significant correlation with droplet digital PCR (ddPCR) and provided a more comprehensive melanoma mutation profile. Our custom panel could be further optimized by replacing amplicons spanning the *TERT* promoter, which did not perform well due to the high GC content. To increase the detection rate to 90% of stage IV melanoma and decrease the sensitivity to 0.1% MAF, we recommend increasing the volume of plasma to 8 mL to achieve minimal recommended cfDNA input and the refinement of poorly performing amplicons. Our panel can also be expanded to include new targetable and treatment resistance mutations to improve the tracking of treatment response and resistance in melanoma patients treated with systemic drug therapies.

## 1. Introduction

The analysis of circulating tumor DNA (ctDNA) is progressively being integrated into routine clinical care to monitor cancer progression, response to therapy, emergence of resistance and to direct therapy [1,2,3,4,5,6]. In melanoma, longitudinal ctDNA assessment predicts overall survival of stage IV melanoma patients treated with either BRAF and MEK inhibitors or immunotherapy [7,8,9,10,11] and the survival of patients with high-risk stage III resected melanoma [12,13,14,15]. ctDNA can also monitor the appearance of treatment-resistant melanoma subclones [7], tumor heterogeneity [16], metabolic tumor burden [17] and differentiate “true progression” from “pseudoprogression” in melanoma patients treated with immunotherapy [18].

Melanoma ctDNA is often detected using single gene assays that monitor a driver mutation that has been previously identified in patient-matched cancer tissue [7,8,12,15,19]. Although next generation sequencing (NGS) can provide an unbiased and comprehensive mutation profile of ctDNA, it is technically challenging due to the limited quantities of highly-fragmented ctDNA [20]. Furthermore, with increased genomic coverage and sequencing error rates in the order of 0.1–1%, whole-exome NGS of ctDNA does not provide the limit of detection (LOD) required to accurately identify low frequency mutations (<1%), which may occur in pre-existing or emerging treatment resistant subclones [16,21]. The use of customized gene mutation panels in NGS of ctDNA can produce significantly lower levels of mutation detection [22], and these panels typically monitor common melanoma driver mutations in *BRAF*, *NRAS* and *KIT*, along with mutations in tumor suppressor genes, such as *TP53*. However, such gene panels do not include many established mutations associated with treatment resistance and do not allow for the discovery and tracking of novel resistance mutations [22]. A commercially-available targeted melanoma panel (UltraSEEK) for ctDNA, which covers 61 mutations in 13 genes with a LOD of 0.1%, has been developed for the study of melanoma disease progression and resistance to systemic treatments [23,24,25]. The UltraSEEK panel is based on mutation detection by mass spectrometry, and, although it showed similar sensitivity to ddPCR in Stage IV melanoma known to carry mutations in plasma, the concordance in the mutations detected was only 88% between these two platforms [24]. Furthermore, in one study the UltraSEEK detection rate for stage IV melanoma was only 66.7% [25]. To date, only one study has employed a targeted custom melanoma NGS panel (950 amplicons over 30 genes) to analyze ctDNA and matched tissue from a cohort (*n* = 24) of stage IV melanoma patients [26]. In this small study, a confirmed driver mutation was identified in 70% of matching plasma samples.

In this study, we developed a melanoma NGS panel for ctDNA analysis based on 123 amplicons covering 30 genes with a predicted detection rate of 90% for cutaneous melanoma and 95% for uveal melanoma [27,28]. The performance of this melanoma mutation panel was evaluated in 74 treatment-naïve stage III and IV melanoma patients to identify whether stage III and IV melanoma could be monitored using a simple blood test.

## 2. Results

### 2.1. Cohort and Sample Characteristics

In total, 91 consecutive patients were recruited between April 2018 and December 2019. Seventeen patients were excluded from analysis as they were subsequently confirmed to have a non-melanoma malignancy (*n* = 3) or the liquid biopsy sample was taken after surgical resection (*n* = 14). Of the evaluable 74 treatment-naïve melanoma patients, 36 (49%) patients had stage III and 38 (51%) had stage IV melanoma (Table 1). The median age of the cohort was 65 years (range 23–89), the majority were male (*n* = 54, 73%) and all but one patient had cutaneous melanoma (uveal melanoma, *n* = 1). Lymph node metastasis were present in 27/36 (75%) stage III patients. Of these, 26/27 (96%) patients had clinically-detectable lymph node metastasis, whereas 1/27 (4%) patients had subclinical lymph node metastases at time of liquid biopsy. The median diameter of the largest lymph node metastases was 28 mm (range 2–81 mm). The remaining nine patients had in-transit metastases only. Of the patients with stage IV disease, 5 patients had M1a, 7 had M1b, 16 had M1c and 10 had M1d disease (with concurrent extracranial metastases in 9/10 patients).

**Figure 1:**
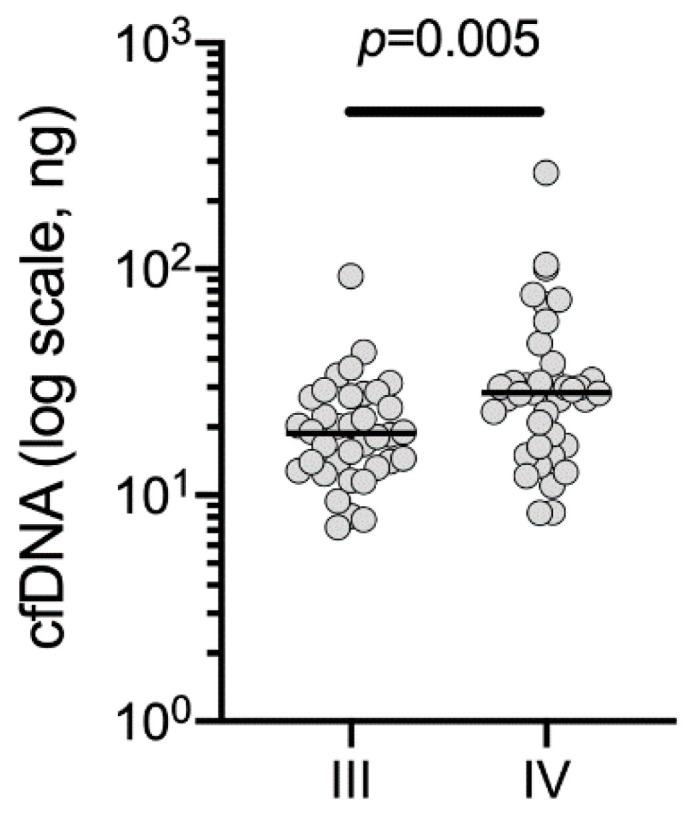
Total yield of cfDNA per patient for stage III vs. stage IV melanoma patients. Mann–Whitney, unpaired, nonparametric *t*-test was performed.

At least 4 mL of plasma was available for 72/74 (97%) patients, and the total recovered quantity of cfDNA ranged from 7.2–266.8 ng (median 22.75 ng). cfDNA quantity was significantly higher in patients with stage IV than stage III disease; median cfDNA was 28.75 ng versus 18.45 ng, respectively (Figure 1). Using a cfDNA threshold for NGS of 20 ng in 16.5 µL (i.e., 1.21 ng/ µL as recommended in the Ion Ampliseq HD library kit user guide), 9/36 (25%) stage III and 19/38 (50%) stage IV patients met this cfDNA threshold (Table 1).

### 2.2. Evaluation of the Performance of the Custom Melanoma Panel

**Figure 2:**
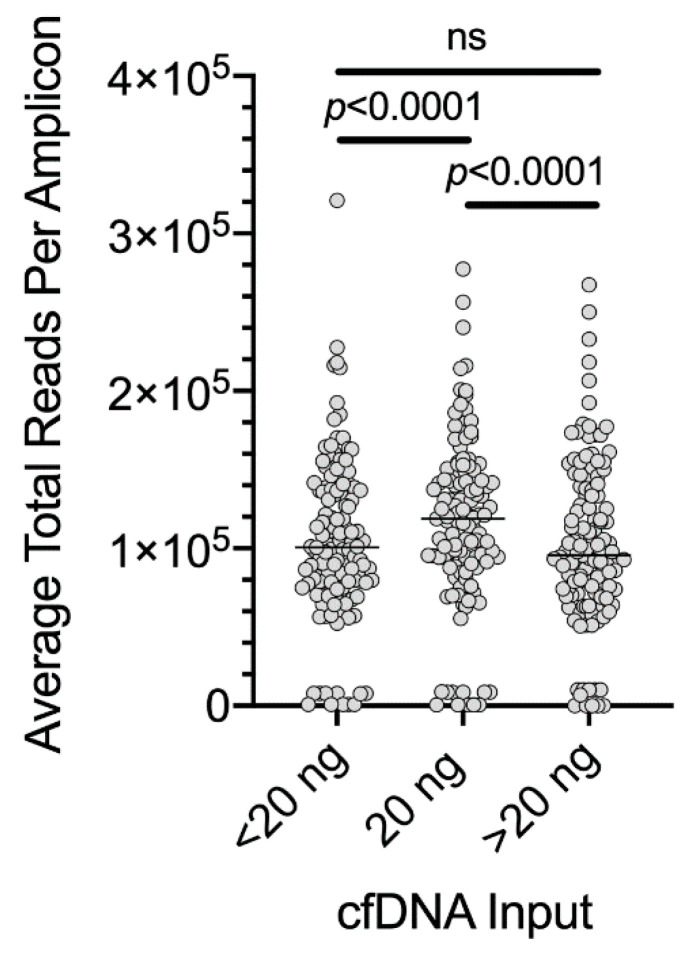
Average total reads obtained for each amplicon in the custom melanoma panel. The input template cfDNA was <20 ng (*n* = 40 samples, range of 3.6–19.1 ng), 20 ng (*n* = 21 samples, range of 19.5–20.5 ng) or >20 ng (*n* = 8 samples, range of 21–42 ng). In each case, seven NGS sample libraries were run on an Ion 550 sequencing chip using the Ion Ampliseq HD workflow. Reading depth data for each amplicon for each patient are shown in Table S2. One-way ANOVA with Tukey’s multiple comparison test was performed. ns, not significant.

**Figure 3:**
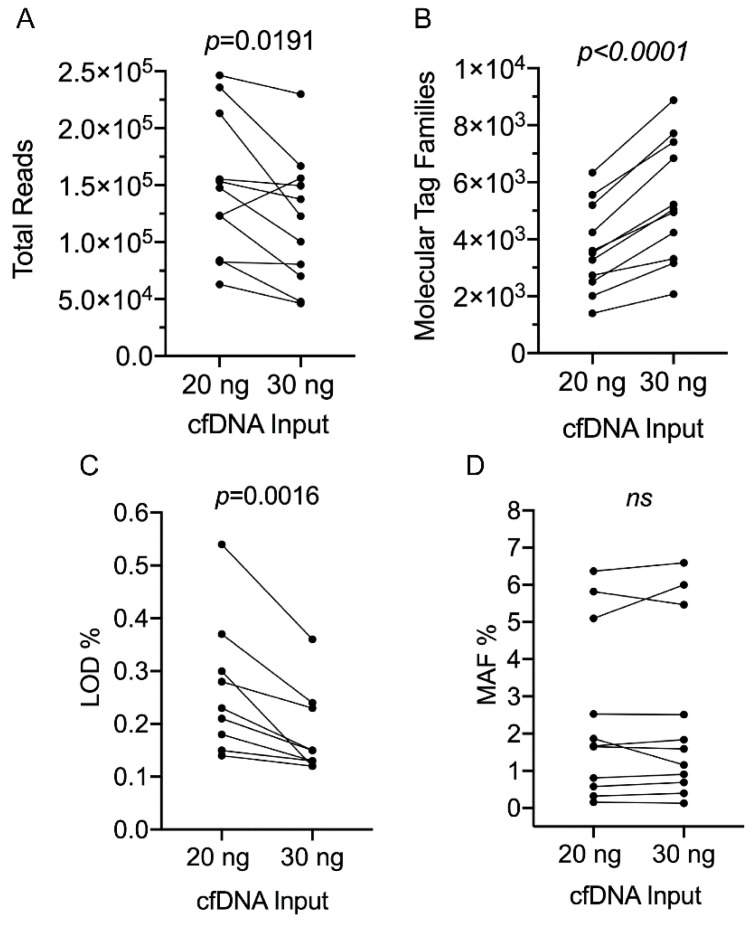
Relationship between sequencing performance, variant detection and cfDNA template input. (**A**–**D**) Pairwise comparison of mutations detected in matched patient samples (refer to Table S3) using either 20 or 30 ng cfDNA template input: (**A**) total reads; (**B**) molecular tag families (defined in materials and methods) per individual amplicon; (**C**) limit of detection (LOD); and (**D**) mutant allele frequency (MAF). Parametric paired *t*-tests were performed. ns, not significant.

The performance of the melanoma panel (Table S1) was initially evaluated based on amplicon reading depth, with an expected ~120,000X median read depth per amplicon according to the capacity of the Ion 550 sequencing chip with seven samples run per chip with the manufacturer’s recommended 20 ng cfDNA input (Figure 2). As expected, reading depth was significantly lower (average~100,000X) when input cfDNA was less than 20 ng, and was also diminished when input cfDNA was increased to >20 ng (Figure 2). This was particularly evident when we compared patient-matched amounts of 20 and 30 ng cfDNA from two melanoma patients and one patient with a non-melanoma malignancy. A pairwise comparison showed a significant decrease in total amplicon reads when cfDNA template was increased to 30 ng (Figure 3A). In contrast, there was a significant increase in the number of molecular tag families (Figure 3B), and this was associated with a significant decrease in LOD (Figure 3C) with no detectable changes in MAF (Figure 3D).

Regardless of the median amplicon sequencing reading depth or coverage (number of unique reads) achieved for the melanoma panel, the relative performance of individual amplicons remained consistent with similar relative read depth regardless of the amount of input template cfDNA (Figure S1). Several amplicons, including the amplicon spanning EIF1AX amino acids 6–13 and the *TERT* promoter amplicons showed consistently lower reading depths relative to other amplicons (Figure S1). As a result, no *EF1AX* or *TERT* promoter mutations were detected in our patient cohort. Mutation calls could not be made for the *TERT* promoter amplicons because no reverse sequencing reads were obtained (data not shown).

The LOD in our panel was analyzed by spiking patient samples with NRAS A59T and Q61K mutations at the MAFs of 1.3%, 0.26% and 0.13% (Horizon standards). The melanoma panel consistently detected both *NRAS* mutations at 1.3% and 0.26% frequency but did not accurately detect the *NRAS* mutations at 0.13% MAF (Table S4). This indicates the LOD for our custom panel was approximately 0.2%.

### 2.3. Identification of Melanoma Mutations Using the Custom Melanoma Panel

**Figure 4:**
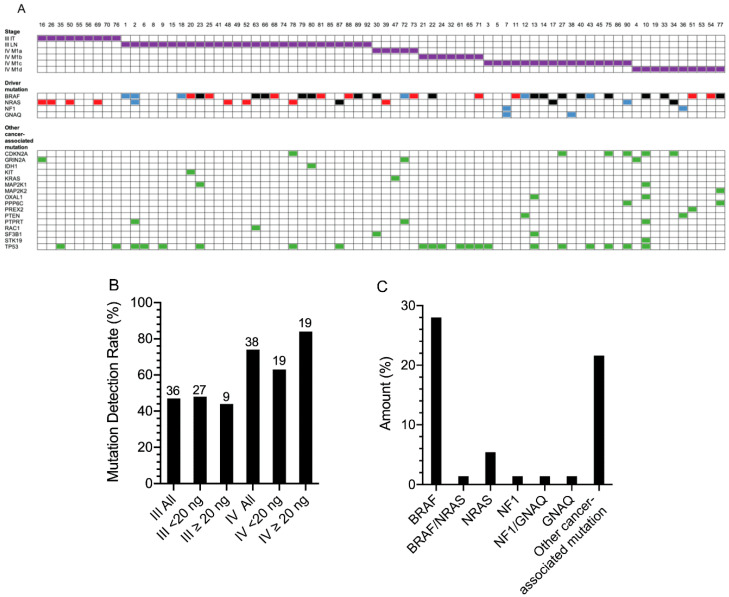
Summary of the melanoma mutation profile and rate of detection based on the custom melanoma panel. (**A**) Comparison of melanoma stage (purple boxes), driver mutation detected by tissue biopsy (red boxes), driver mutation detected by liquid biopsy (blue boxes), driver mutation detected by both liquid and tissue biopsy (black boxes) and other cancer-associated mutations (green boxes) identified across the cohort of 74 melanoma patients. Numbers represent patient sample number. IT, in-transit; LN, lymph node. For details on specific gene mutations and complete NGS data, refer to Tables S5 and S6. (**B**) Comparison of mutation detection rate for melanoma mutations based on the level of cfDNA used for NGS. Total sample number is shown above each histogram. For details on stage, cfDNA input and melanoma driver or other cancer-associated mutation detected, refer to Table S5. (**C**) Distribution of melanoma driver mutation (BRAF, GNAQ, NF1 and NRAS) or other cancer-associated mutation (only including those which lacked a BRAF, GNAQ, NF1 or NRAS driver mutation). Amount indicates percent of total melanoma cohort (*n* = 74).

Melanoma mutations, either driver (*BRAF*, *NRAS*, *NF1*, *GNAQ;* identified in *n* = 29 patients) or other cancer-associated mutation (identified in *n* = 16 patients; defined in Table S5), were identified in 45/74 (61%) melanoma patients using our custom melanoma panel, 17/36 (47%) for stage III and 28/38 (74%) for stage IV (Figure 4A). When tumor tissue mutation testing was available, a matched ctDNA and tumor driver mutation was confirmed in 19/19 patients (Figure 4A). ctDNA melanoma mutation detectability was also stratified according to disease distribution and stage. For stage III patients, ctDNA melanoma mutation detectability was 14/27 (52%) for patients with lymph node metastasis and 3/9 (33%) for patients with in-transit metastases only, without lymph node metastases (Figure 4A). For stage IV patients, detectability was 3/5 (60%) for M1a, 6/7 (86%) for M1b, 12/16 (75%) for M1c and 7/10 (70%) for M1d melanoma (Figure 4A). Increasing input cfDNA increased mutation detection in stage IV patients but not in stage III patients. In particular, whereas only 12/19 (63%) stage IV patients had detectable mutations with input cfDNA <20 ng, this increased to 16/19 (84%) mutations detected with input cfDNA ≥20 ng (maximum of 30 ng) (Figure 4B).

We were particularly interested in the 10 stage IV patients who did not have a detectable driver or other cancer-associated mutation in the ctDNA using our custom melanoma panel. Nine of these patients had driver mutation data derived from the tumor specimen: 5/9 patients had *BRAF*/*NRAS*/*KIT* wild type melanoma, whereas 4/9 patients had *BRAF* or *NRAS* driver mutations identified in the tumor. In particular, NRAS Q61L and BRAF V600E mutations were identified in two patients with M1a disease, while BRAF V600E and BRAF D594N mutations were found in one patient each with M1c and M1d disease, respectively. Both patients with M1c and M1d disease had very low volume disease: the M1c patient had 6-mm lung and 23-mm peritoneal metastases and the M1d patient had a brain metastasis and a subcentimeter solitary lung metastasis.

We identified *BRAF* mutations in 21/74 (28%) patients, *NRAS* mutations in 4/74 (5.4%) patients, *NF1* mutations in 1/74 (1.4%) patients and *GNAQ* mutations in 1/74 (uveal melanoma) (1.4%) patients, as well as *BRAF*/*NRAS* (1/74; 1.4%) and *GNAQ*/*NF1* (1/74; 1.4%) double mutations (Figure 4C). Other cancer-associated mutations, including *TP53*, *CDKN2A* and *PTEN* alterations, were also identified in another 16/74 (22%) patients (Figure 4C). Other mutations were identified in 20 patients who did not have a detectable melanoma driver or other cancer-associated mutation (Table S5). The majority of these mutations were detected at low MAFs and were below the LOD and thus would require further validation. Only 4/20 patients had other ctDNA-associated mutations detected at a MAF of >0.2% (GRIN2A P1171L, RAC1 A27P, STK19 T80N and TP53 V173M).

**Figure 5:**
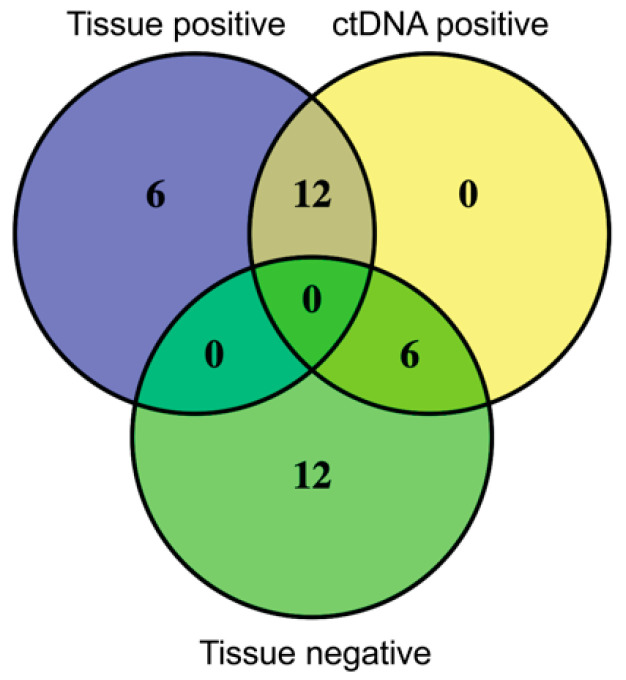
Concordance between tissue and ctDNA driver mutations for stage IV melanoma patients (*n* = 36). Values shown represent number of patients.

The concordance between tissue and ctDNA driver mutations was performed in 36 of the 38 stage IV patients who had undergone tissue mutation analysis (three patients had VE1 immunohistochemistry only) (Table S7). In 18/36 patients, a melanoma driver mutation was detected in the tissue specimen and a matching ctDNA mutation was detected in 12 of these patients (66.7%) (Figure 5). In Patients 13 and 33, an additional rare BRAF driver mutation was identified in the ctDNA sample (Table S7). In the 18 patients with no driver mutation detected in the melanoma tissue biopsy, six patients had a driver mutation identified in the ctDNA (Figure 5), including three patients with rare BRAF kinase domain mutations (BRAF G466A, BRAF G469A and BRAF T599dup) and one patient with a predominant NF1 R1241* nonsense mutation (MAF 27.65%) and a low frequency GNAQ R183Q mutation (MAF 0.71%) (Table S7). Cancer-associated mutations were also identified in the ctDNA of 9/36 patients (Table S7), and, although these were not validated in this study, it is worth noting that six of these nine patients (67%) had no driver mutation identified in the tissue biopsy.

### 2.4. Validation of Custom Melanoma Panel

**Figure 6:**
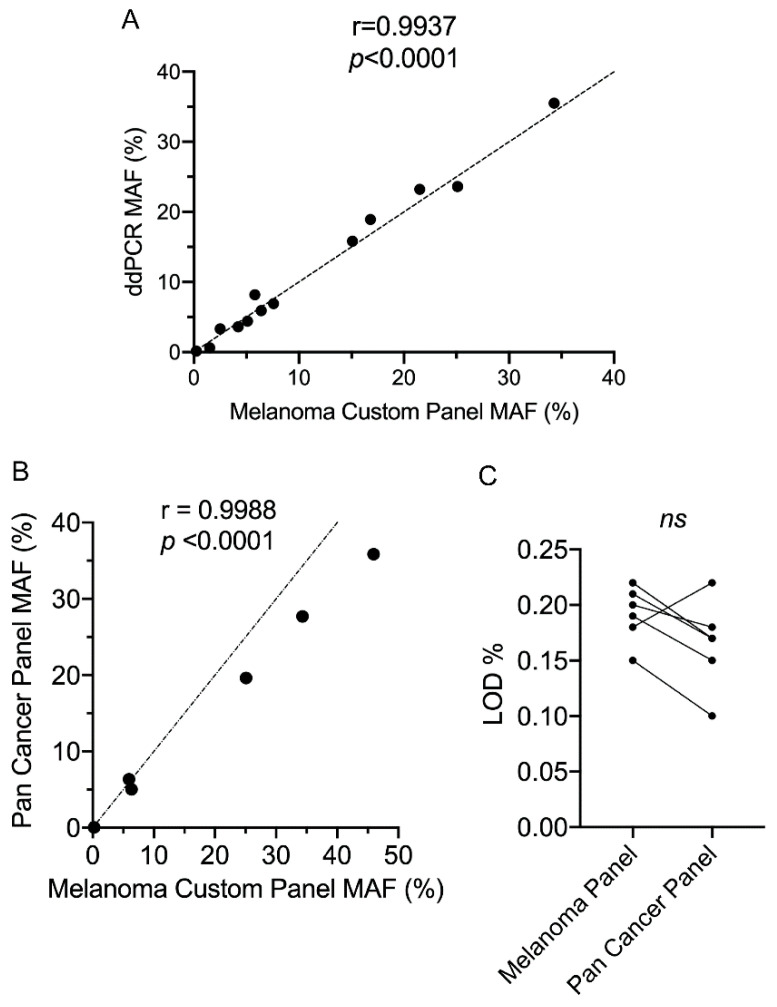
Validation of the custom melanoma panel. (**A**) Correlation of mutant allele frequency (MAF) determined by the melanoma custom panel versus ddPCR analysis. (**B**,**C**) Comparison of custom melanoma and Oncomine pan cancer targeted NGS panels. (**B**) Correlation of MAF based on mutations detected by both panels. (**C**) Pairwise comparisons of limit of detection (LOD) for matched mutations detected using both assays. For patient samples tested along with NGS and ddPCR data refer to Tables S8 and S9. Pearson correlation coefficient analysis and parametric paired *t*-test were performed.

We initially analyzed the performance of our custom melanoma panel by validating driver mutations identified in the ctDNA of 13 selected patients. These patients were chosen based on the availability of ddPCR probes and sufficient cfDNA template. Using ddPCR, we validated all 13 driver mutations and confirmed there was a significant correlation in the MAFs generated by ddPCR and our custom NGS assay (Figure 6A).

Next we compared the performance of our custom melanoma panel with the 52-gene Thermofisher Oncomine pan cancer panel. For this analysis, five patients from our melanoma cohort plus three patients from the non-melanoma patients were selected based on ctDNA availability. There was significant correlation in the MAF for each identified mutation between the two cancer panels (Figure 6B). We also observed that the LOD of driver and cancer-associated mutations was not significantly different between the two cancer panels, although the custom panel did not reach the specified value of 0.1% (Figure 6C).

### 2.5. TERT Promoter Mutations

We also took the opportunity to assess *TERT* promoter mutations using ddPCR in 10 patients. The −124 C > T and −146 C > T [29,30] mutations were not detected using our custom melanoma panel and −124 C > T was identified using ddPCR in patients 34 and 56 at a MAF of 0.8% and 0.15%, respectively (Table S10). Patient 34 also had a NRAS Q61K mutation, confirmed by both tissue and ctDNA NGS. The manual evaluation of the sequencing Bam files from our custom panel confirmed the presence of the −124 C > T *TERT* mutation in patients 34 and 56 with MAFs of 1.1% and 0.17%, respectively. A *TERT* promoter mutation, −146 C > T, was found in patient 88 at a MAF of 0.33% using ddPCR, although this was with only one positive droplet in the mutant channel. This mutation was not detected in the NGS sequencing Bam file generated using the custom melanoma panel.

## 3. Discussion

There are now many commercially available tumor-specific NGS panels for liquid biopsy, for cancers such as lung, breast and colorectal. In addition, there are several pan-cancer liquid biopsy NGS panels which provide the flexibility of covering several cancers but at a lower depth of coverage for known tumor-specific driver mutations [22]. To the best of our knowledge, there is only one cutaneous melanoma specific panel for liquid biopsy on the market [23,24] which includes only 61 variants across 16 genes. In this study, we developed a broader melanoma-specific panel for liquid biopsy with a higher coverage of mutations for both cutaneous and uveal melanoma.

We developed and tested a custom melanoma NGS panel, based on Ampliseq HD, on a cohort of 74 melanoma patients which had a theoretical coverage of 90% of cutaneous melanoma and 95% of uveal melanoma [27,28]. With an optimal cfDNA input of 20 ng, we reached a 74% detection rate, based on detection of driver or cancer-associated mutation, of metastatic disease (stage III and IV patients), which increased to an 84% detection rate in stage IV melanoma patients.

To improve the performance of this panel, we plan to remove or improve underperforming amplicons which take up sequencing reads, such as those covering *TERT* promoter (discussed below), *DDX3X* and *EIF1AX*. Both *DDX3X* and *EIF1AX* mutations account for 10% of melanomas and they generally co-occur with *NRAS* mutations [27,28]. Although their removal from the panel should have no significant impact on the overall detection rate of mutations in ctDNA, these mutations may have prognostic value [31,32,33] and improving amplicon design will be explored. Another option to improve the detection rate of our panel would be to increase the mutation analysis coverage of *NF1*. Based on ctDNA, just 3% of this cohort had *NF1* mutations, which is significantly lower than the TCGA value of 10% for cutaneous melanoma [27,28]. Given *NF1* exons span 8520 bp and sequencing amplicons are typically less than 100 bp, it would not be feasible to cover the complete *NF1* gene, but the select inclusion of 10 additional amplicons would cover the majority of *NF1* mutations identified in *BRAF*/*NRAS* wild-type melanoma patients and should increase our coverage to ~90% [27,28].

The MAF of a BRAF G469A mutation based on ctDNA using our custom panel was found to be <0.2% in two stage IV melanoma patients. These same mutations were not detected in the matching tissue biopsy. We presume that these mutations are subclonal and therefore may not be represented (or exist at very low frequency) in the matching tissue biopsy.

Based on MAF, our custom melanoma panel showed a correlation of 0.994 when compared to ddPCR analysis and 0.9988 when compared to analysis with an off-the-shelf pan cancer NGS panel. We found that our custom panel did not reach the expected LOD of 0.1% (based on the sensitivity of ddPCR and off-the-shelf panels [22]). Typically, for the Ampliseq HD NGS workflow used with our custom panel, ≥3000 molecular tag families/amplicon are required to achieve a LOD of 0.1% (personal communication from Thermofisher). The number of molecular tag families present across the *NRAS* amplicon, present in Horizon reference standard, was found to be in the range 1400–1800 for our custom melanoma panel. This would explain why for our custom melanoma panel, when using the optimum 20 ng of template, we only achieved a LOD of ~0.2% when testing the Horizon reference standards.

Many studies using NGS panels do not report the LOD relative to MAF. In our study, we comprehensively reported both values to emphasize the care required when calling mutations with low MAF (<0.5%) which may be less than the LOD. We found that increasing cfDNA directly correlated with increasing molecular tag families in our sequencing libraries which inversely correlated with the LOD. Unexpectedly, cfDNA input above 20 ng produced lower reading depths, although these data should be interpreted with caution as they were derived from a small number of sequencing runs. Collectively, our data indicate that increased plasma volumes would be expected to lower the LOD and is worth considering, especially as there is a linear recovery of cfDNA up to 17.5 mL of input plasma with the extraction kit used here [34]. Given that 50% of stage IV patients had less than the recommended cfDNA input of 20 ng (we observed an increase in detection rate from 63% (<20 ng cfDNA) to 84% (≥20 ng cfDNA) for stage IV) increasing the amount of available cfDNA, through processing 8 mL of plasma, would also increase our detection rate in stage IV melanoma.

We found that 28% (21/74) of our patient cohort had *TP53* mutations. Some of these p53 mutations have been shown to be a result of clonal hematopoiesis. This is a process whereby expansion of blood cell clones occurs due to advantageous somatic mutations, which often accumulate with age, and may contribute to hematological malignancies [35,36,37,38]. Having a broad NGS panel, that can simultaneously detect multiple low frequency variants becomes critical in defining the significance of circulating mutations.

The *TERT* promoter mutations −124 C > T and −146 C > T tend to be mutually exclusive and are reported to account for anywhere from 32% (based on NGS analysis of tissue [26]) to 68–78% (based on ddPCR analysis of tissue [39,40]) of all melanomas. As already highlighted, *TERT* promoter amplicons did not perform well in our custom melanoma panel, presumably due to the high GC content of around 80%, and we did not detect any *TERT* promoter mutations in our cohort. A similar observation has been reported using a custom melanoma NGS panel analysis of plasma ctDNA from stage IV melanoma patients [26]. The Guardant360 pan cancer NGS panel (covers 73 genes) developed by Guardant Health has successfully detected *TERT* promoter mutations in ctDNA from a number of cancers, although melanoma was not included [41,42]. The methodology for achieving successful sequencing of the *TERT* promoter was not disclosed in these published studies. Importantly, *TERT* promoter mutations are detectable by ddPCR and the assay sensitivity can be enhanced by incorporation of 7-deaza-dGTP [26,39,43]. We therefore also incorporated 7-deaza-dGTP in our ddPCR assay to analyze ctDNA from 10 of our patient cohort and found 30% were positive for *TERT* promoter mutations. The MAF of these *TERT* promoter mutations were low (in the range 0.15–0.8%) compared to values of 2–20% previously reported for a cohort of stage IV melanoma patients [26].

Ideally, detection of *TERT* promoter mutations needs to be included in any future workflow to maximize detection of melanoma based on ctDNA. Given the difficulty encountered with sequencing of the TERT promoter using NGS, an alternative custom melanoma panel based on ddPCR could be developed. This panel could include multiplex screening with primer/probes for detection of BRAF V600E/K/R, NRAS G12A/C/D/S/V, NRAS G13D/R/V, NRAS Q61H/K/L/R along with pooled primer/probes for *TERT* promoter mutations −124 C > T and −146 C > T. This concept of following *BRAF*, *NRAS* and *TERT* in the context of melanoma in a multiplex assay has been explored in a recent study [40]. This custom ddPCR panel would have a predicted coverage of ~80% of cutaneous melanomas based on the skin cutaneous melanoma TCGA dataset which gives a *BRAF*/*NRAS* coverage of ~70% [27,28] and the fact that *TERT* promoter mutations are found in around ~29% of *BRAF* wild-type melanoma patients [39]. Analysis of *NRAS* and *BRAF* mutations in stage IV melanoma patients by single reaction ddPCR has shown an overall detection rate of 73% [7]. Such a single multiplex ddPCR reaction is feasible given the recent release of new digital PCR platforms from both Bio-Rad and Qiagen with multiplexing capabilities due to the incorporation of four color channels. Such a ddPCR panel, with lower cost, turnaround time of 1 day and requirement for less cfDNA (<10 ng) than the NGS assay (>20 ng), could be used as a first pass for detection of melanoma mutations using ctDNA. Given the ddPCR assay requires less cfDNA template it would also therefore require less plasma (typically 2 mL). Those melanoma patients who fail detection with the custom ddPCR panel could then be analyzed using our optimized (~90% coverage) custom melanoma NGS panel. The NGS assay would require 4–8 mL of plasma to obtain sufficient cfDNA template with the precise volume of plasma determined by the initial cfDNA extraction used for ddPCR.

## 4. Materials and Methods

### 4.1. Human Melanoma Samples

The fresh-frozen tissue and blood samples used in the current study were obtained from the Melanoma Institute Australia biospecimen bank with written informed patient consent and institutional review board approval (Sydney Local Health District Human Research Ethics Committee, Protocol No. X15–0454 and HREC/11/RPAH/444). The Oncofocus panel (Agena Bioscience, San Diego, CA, USA) was used for detection of melanoma-associated *BRAF*, *NRAS*, *KRAS* and *KIT* variants in paired tissue samples [44,45]. Immunohistochemistry to detect BRAF V600E using VE1 monoclonal antibody (Abcam, Cambridge, UK) was performed as previously described [46].

Blood (10 mL) was collected in EDTA tubes (Becton Dickinson, Franklin Lakes, NJ, USA) and processed within 4 h from blood draw. Tubes were spun at 800 g for 15 min at room temperature. Plasma was then removed into new 15 mL tubes without disturbing the buffy coat and respun at 1600 *g* for 10 min at room temperature to remove cellular debris. Plasma was stored in 1–2 mL aliquots at −80 °C.

### 4.2. Purification of Circulating Free (DNA) (cfDNA) from Plasma

Plasma cfDNA was purified using the QIAamp circulating nucleic acid kit (Qiagen, Hilden, Germany) according to the manufacturer’s instructions. cfDNA was purified from 4 mL of plasma except for Samples 13, 27, 69, 70, 76 and 80 for which 4.5–5 mL of plasma were used and Sample 34 for which 3.5 mL of plasma were used. The final elution volume was 25 μL in all cases. cfDNA was subsequently quantified using a Qubit dsDNA high sensitivity assay kit and a Qubit fluorometer 3 (Life Technologies, Carlsbad, CA, USA), according to the manufacturer’s instructions.

### 4.3. Custom Melanoma Gene Panel for Targeted NGS of cfDNA

An Ion Ampliseq HD made-to-order melanoma gene panel consisting of individual forward and reverse primers in 384 well-format to produce 123 amplicons (Table S1) was obtained from Life Technologies (Carlsbad, CA, USA). The panel covers 30 gene targets (Table S1 [30,47,48,49,50,51,52,53]) and was predicted to cover 90% of cutaneous melanoma (skin cutaneous melanoma TCGA dataset) and 95% of uveal melanoma patient mutations (uveal melanoma TCGA dataset [27,28]). The panel covers nucleotide variants which give rise to melanoma-associated amino acid changes across 29 gene targets [47,48,49,50,51,52], as well as 6 melanoma-associated nucleotide variants in the promoter region of the *TERT* gene [30,53].

In addition to running cfDNA from melanoma patients, analysis of the multiplex I cfDNA reference standard set (Cat#HD780; Horizon Discovery, Waterbeach, UK) was also performed using the Ion Ampliseq HD custom panel. We generated a 0.26% MAF reference standard by diluting the provided 1.3% MAF reference standard with the appropriate amount of the 100% wild-type reference standard. Details of library amplification and sequencing workflows for both the custom melanoma panel and an Oncomine pan cancer panel are provided in Supplementary Methods.

### 4.4. ddPCR Analysis of ctDNA from Plasma

The copy number of ctDNA per mL of plasma was determined using the QX200 droplet digital PCR (ddPCR) (Bio-Rad, Hercules, CA, USA) system to detect tumor-associated BRAF E586K, V600E/K, K601E, GNAQ R183C, Q209P or NRAS Q61K mutations, as previously described [8]. ddPCR mutation assays for BRAF E586K, K601E and GNAQ R183C were kindly provided by Elin Gray (Edith Cowan University) while the remainder were obtained from Bio-Rad (Hercules, CA, USA). *TERT* promoter mutations −124 C > T and −146 C > T were identified using ddPCR expert design assays dHsaEXD20945488 (*TERT* C228T_88) and dHsaEXD85215261 (*TERT* C250T_88) [54] (Bio-Rad, Hercules, CA, USA), according to the manufacturer’s instructions. The *TERT* promoter assays were optimized by inclusion of 200 µM 7-deaza-dGTP (New England Biolabs, Ipswich, MA, USA), as previously described [43]. The DNA copy number/mL of plasma for mutant and wild-type circulating DNA species was determined with Quantasoft software version 1.7.4 (Bio-Rad, Hercules, CA, USA) using a manual threshold setting. The minimum number of positive droplets for calling a mutation was set at two.

### 4.5. Statistical Analysis

One-way ANOVA with Geisser–Greenhouse correction and Tukey’s multiple comparison test, comparing mean of each column with mean of every other column, were performed using Graphpad Prism version 8.3.1. Pearson correlation coefficient analysis, parametric paired *t*-tests and a Mann–Whitney unpaired, non-parametric *t*-test were also performed using Graphpad Prism version 8.3.1. Hierarchical clustering using Euclidean distance with complete linkage was undertaken using Morpheus (https://software.broadinstitute.org/morpheus). Venn diagrams were generated using the interactive online tool Venny 2.1 (http://bioinfogp.cnb.csic.es/tools/venny/index.html).

## 5. Conclusions

In this study, we developed a custom melanoma NGS panel. Analysis of a cohort of treatment-naïve melanoma patients revealed that sensitivity of ctDNA detection was influenced by the amount of cfDNA input and extracranial disease burden. With refinement, our panel will prove particularly useful in detecting tumor heterogeneity, potentially new targetable mutations and tracking treatment response and resistance in melanoma patients treated with systemic drug therapies. The development of a robust ctDNA profiling process will also reduce the need for invasive tissue biopsies and enable longitudinal, real-time monitoring of patients.

## Figures and Tables

**Table 1:** Summary of patient cohort.

Characteristics	Stage III (*n* = 36)	Stage IV (*n* = 38)
Age (years)		
Median (range)	66 (32–89)	61 (23–88)
Sex, n (%)		
Male	26 (72)	28 (74)
Female	10 (28)	10 (26)
Disease distribution, n (%)		
LN metastases	27 (75)	
IT disease only	9 (25)	
AJCC M stage, n (%)		
M1a		5 (13)
M1b		7 (18)
M1c		16 (42)
M1d		10 (26)
Tissue mutation profile, n (%)		
*BRAF*	11 (31)	15 (39)
*NRAS*	8 (22)	3 (8)
*BRAF* WT ^1^	7 (19)	3 (8)
*BRAF/NRAS/KIT* WT ^2^	5 (14)	15 (39)
Not performed	5 (14)	2 (5)
cfDNA, n (%)		
Threshold for NGS (20 ng/16.5 µL)	9 (25)	19 (50)

^1^ VE1 immunohistochemistry negative for BRAF V600E. ^2^ No mutation found on molecular testing. Abbreviations: NGS, next generation sequencing; LN, lymph node; IT, in-transit.

## Supplementary Materials

The following are available online at https://www.mdpi.com/2072-6694/12/8/2228/s1. Supplementary methods; Figure S1: Summary amplicon performance; Table S1: Custom melanoma amplicons; Table S2: Summary total reads; Table S3: Summary matched patient samples; Table S4: Panel calibration; Table S5: Patient mutations identified; Table S6: Summary patient NGS; Table S7: Stage IV Concordance; Table S8: ddPCR vs. NGS; Table S9: Custom vs. pan; Table S10: TERT ddPCR.
