# Supplementary material for: Design and Testing of a Custom Melanoma Next Generation Sequencing Panel for Analysis of Circulating Tumor DNA
Source: Cancers (Basel). 2020 Aug 10;12(8):2228. doi: 10.3390/cancers12082228 (PMC7465941; doi:10.3390/cancers12082228)
Supplement: Supplementary file 1 [file cancers-12-02228-s001.zip › CancersSupplementaryMethods.docx]

**Supplementary Methods**

**Custom melanoma gene panel library amplification and sequencing workflow**

10X (500 nM) forward and reverse primer pools were generated by combining 2 μl of each primer (provided at a concentration of 307 μM) and adding low TE (10 mM Tris, pH 8.0; 0.1 mM EDTA) to a final volume of 1.228 ml. DNA target amplification, using cfDNA as template, partial digestion and barcoding of amplicons was performed according to the manufacturer’s protocol (Ion Ampliseq HD library kit user guide; Life Technologies, Carlsbad, CA, USA). The barcoding set consisted of AmpliSeq HD Dual Barcode Kit 1–24 (Life Technologies, Carlsbad, CA, USA).

Amplified libraries were then subjected to two rounds of purification using Agencourt AMPure XP beads (Beckman Coulter, Brea, CA, USA) according to the Ion Ampliseq HD library kit user guide. The protocol was modified to reduce bead drying time to 4 minutes for both rounds of purification and eluting into a final volume of 40 μl of low TE for the second round of purification.

Purified libraries were then quantified by qPCR using an Ion Library TaqMan Quantitation Kit (Life Technologies, Carlsbad, CA, USA) according to the Ion Ampliseq HD library kit user guide. A 1:100 dilution of each sample library was analysed in each case. qPCR was performed using a ViiA 7 real-time PCR system and a 96 well fast reaction plate (Life Technologies, Carlsbad, CA, USA). Individual barcoded libraries were diluted to a final concentration of 50 pM in nuclease free water using Eppendorf DNA LoBind microcentrifuge tubes (Sigma-Aldrich, St. Louis, MO, USA). Typically for each sequencing chip, equal volumes (5 μl) of 7 diluted library samples were combined on ice. Subsequently, 25 μl of the pooled libraries were used for template preparation and chip loading. Any remaining undiluted libraries were stored at -30^o^C.

The sequencing workflow consisted of template preparation and sequencing chip loading on an Ion Chef instrument (Life Technologies, Carlsbad, CA, USA), sequencing on an Ion GeneStudio S5 plus system (Life Technologies, Carlsbad, CA, USA) followed by data analysis. Planned sequencing runs were based on the AmpliSeq HD for Liquid Biopsy - DNA template using Torrent Suite Software version 5.12.1. Unless otherwise specified the kits used for each planned sequencing run were an Ion 550 kit-Chef (Life Technologies, Carlsbad, CA, USA), Ion S5 sequencing kit (Life Technologies, Carlsbad, CA, USA) and Ion 550 chip (Life Technologies, Carlsbad, CA, USA). In all sequencing runs 500 flows were chosen. Sequencing data was automatically uploaded to Ion reporter software version 5.10 for variant annotation using the Ampliseq HD for liquid biopsy w2.1-DNA-Single Sample workflow (which was subsequently modified to filter out off-target amplifications in KRAS which results in false positive mutations V7A, G13S and G15S). Parameters for molecular tag families were the default settings of a minimum 3 reads, containing at least one read in each direction, with the same unique identifier (UID) required to form a functional molecular tag family and a minimum of 3 functional molecular tag families supporting a variant to make a call. Subsequently Ion reporter variant analysis files were uploaded to Oncomine knowledgebase reporter software version 3.2.3 r.7c8675e which provides a variant summary including highlighting melanoma driver mutations.

**Pan cancer panel for targeted NGS of cfDNA**

An Oncomine pan cancer cell free assay was used according to the manufacturer’s protocol (Life Technologies, Carlsbad, CA, USA). The exception being that the step involving reverse transcription of total cell-free nucleic acid was omitted. A total of 8 samples consisting of 20 ng of cfDNA were used as template in the assay. Each purified library was quantified by qPCR using an Ion Library TaqMan Quantitation Kit, a ViiA 7 real-time PCR system and a 96 well fast reaction plate.

Individual barcoded libraries were diluted to a final concentration of 50 pM in nuclease free water using Eppendorf DNA LoBind microcentrifuge tubes. For each Ion 550 chip to be run, equal volumes (10 μl) of 4 diluted library samples were combined on ice. Subsequently, 25 μl of the pooled libraries were used for template preparation and chip loading. Any remaining undiluted libraries were stored at -30^o^C. The sequencing workflow was as described for the Ion Ampliseq HD custom panel. The exceptions being the template chosen in the Torrent suite software was Oncomine TagSeq 550 Liquid Biopsy before uploading to the Ion Reporter workflow Oncomine TagSeq Pan Cancer Liquid Biopsy w2.1 - Single Sample.
