# Supplementary figures and images for: Design and Testing of a Custom Melanoma Next Generation Sequencing Panel for Analysis of Circulating Tumor DNA
Source: Cancers (Basel). 2020 Aug 10;12(8):2228. doi: 10.3390/cancers12082228 (PMC7465941; doi:10.3390/cancers12082228)

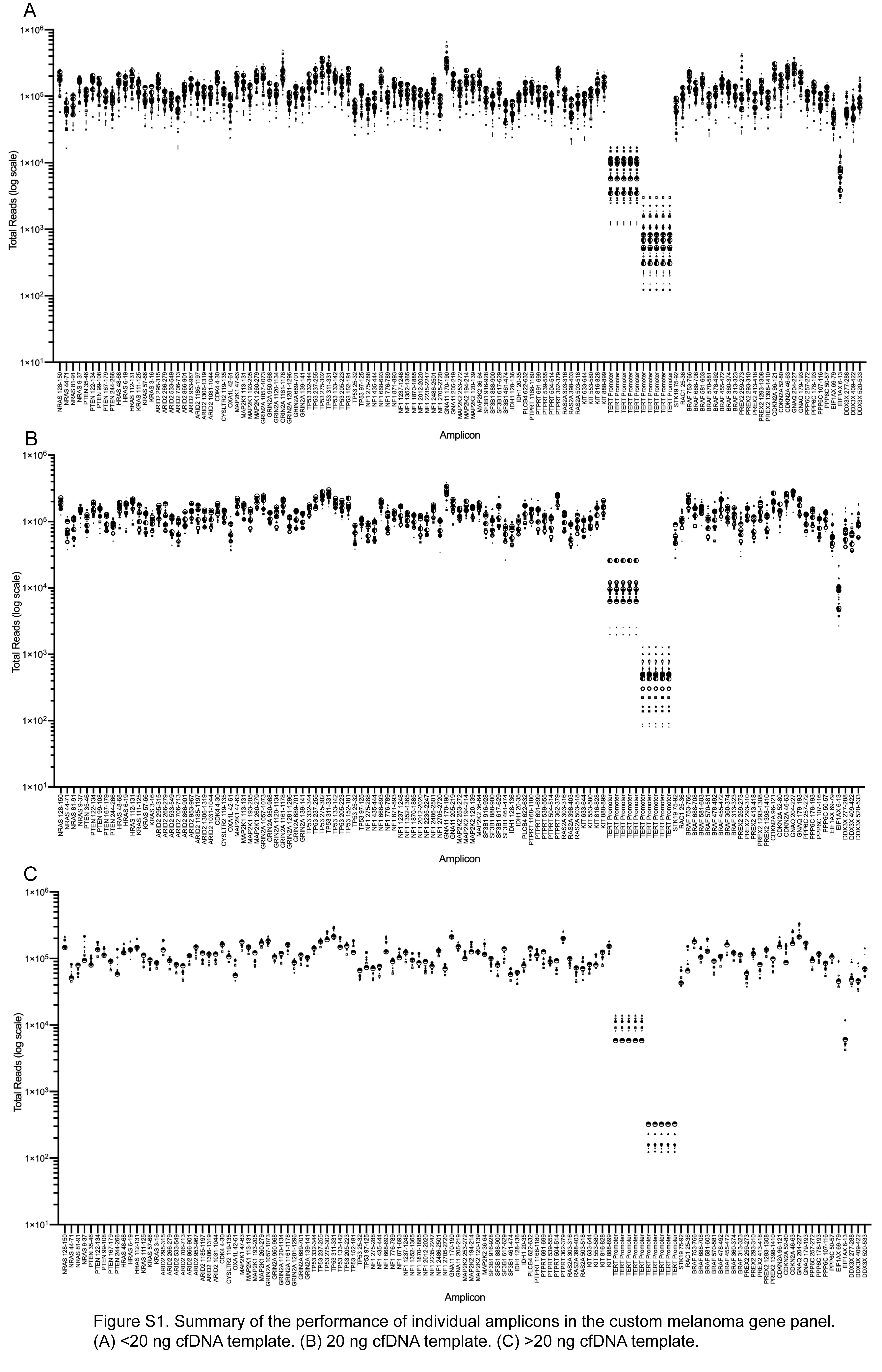

Supplement: Supplementary file 1 [file cancers-12-02228-s001.zip › FigureS1SummaryAmpliconPerformance.tif]
